# Cell-Free Adipose Liquid Extract Combined with Vascularized Lymph Node Transfer for Treating Lymphedema: Implications for Human Therapy

**DOI:** 10.1007/s13770-026-00829-x

**Published:** 2026-07-21

**Authors:** Lingli Jiang, Sanhong Yang, Fang Zhang, Fang Qi, Wenjie Pan, Anna Chiarini, Ilaria Dal Prà, Ubaldo Armato, Daniele De Santis, Hai Li, Shune Xiao, Chengliang Deng

**Affiliations:** 1 Department of Burns and Plastic Surgery, Affiliated Hospital of Zunyi Medical University, Zunyi, Guizhou China; 2 Department of Plastic and Aesthetic Surgery, The People’s Hospital of Baoan Shenzhen (The Second Affiliated Hospital of Shenzhen University), Shenzhen, Guangdong China; 3 Maxillofacial Surgery & Odontostomatology Section, Department of Surgery, Dentistry, Paediatrics & Gynaecology, University of Verona Medical School, 37134 Verona, Italy

**Keywords:** Secondary lymphedema, Vascularized lymph node transfer, Cell-free therapy, Adipose liquid extract, High endothelial venule

## Abstract

**Background:**

To evaluate whether the application of cell-free adipose liquid extract (ALE) synergistically enhances the therapeutic efficacy of vascularized lymph node transfer (VLNT) by reconstructing the lymphatic-vascular network and restoring the lymph node immune niche in a rat lymphedema model.

**Methods:**

A rat hindlimb lymphedema model was established involving popliteal lymph node excision. Animals were randomized into four groups: Control, ALE only, VLNT only, and VLNT combined with ALE (VLNT + ALE). ALE was prepared using a mechanical emulsification and filtration protocol. Edema resolution was monitored by limb circumference. Lymphatic drainage function was visualized via indocyanine green (ICG) lymphography. Histological assessments included Masson’s trichrome and immunofluorescence for LYVE-1 (lymphangiogenesis), CD31 (angiogenesis), and MECA-79 (high endothelial venules, HEVs) to evaluate structural and functional regeneration.

**Results:**

The VLNT + ALE group achieved the most rapid and significant edema regression compared to VLNT or ALE alone. ICG lymphography revealed that the combined therapy orchestrated the formation of continuous, linear lymphatic channels, effectively eliminating dermal backflow. Histologically, ALE treatment significantly increased the density of both LYVE-1^+^ lymphatic vessels and CD31^+^ blood vessels, suggesting a dual-regenerative effect on the microenvironment. ALE directly promoted tube formation in human lymphatic endothelial cells (HLECs), confirming its pro-lymphangiogenic activity in vitro. Crucially, within the transplanted lymph nodes, ALE treatment restored the expression of MECA-79^+^ HEVs to levels comparable to healthy young nodes. This indicates that ALE not only supports graft survival but also preserves the essential lymphoid microarchitecture required for immune surveillance.

**Conclusions:**

Cell-free ALE synergistically enhances the therapeutic efficacy of VLNT by promoting vascular-lymphatic coupling and restoring the functional immune niche of transplanted nodes. As a readily available, safe, and cell-free biologic adjuvant, ALE represents a promising translational strategy to optimize surgical outcomes in secondary lymphedema.

## Introduction

Secondary lymphedema is a chronic, progressive disorder resulting from impaired lymphatic drainage, commonly occurring after oncologic treatments such as lymphadenectomy and radiotherapy for breast or gynecologic malignancies [1, 2]. It is the clinically predominant form of lymphedema, affecting roughly 1 in 1000 individuals, and about 20% of breast cancer survivors. It is characterized by the accumulation of protein-rich lymphatic fluid within the interstitial tissue, accompanied by chronic inflammation, adipose tissue deposition, and fibrosis [3–5], which altogether cause pain, heaviness, limited mobility, recurrent infections, and skin changes. Various physical, psychological, and social sequelae associate with lymphedema. Thus, patients’ quality of life is severely impaired. As a progressive, intractable condition, secondary lymphedema has no definitive cure, making it a disabling disease and a major unmet challenge in rehabilitation medicine.

Conservative therapy such as complex decongestive physiotherapy with manual drainage, bandaging, compression garments, exercise, etc. remains the first-line treatment, but it typically provides only symptomatic relief and cannot restore normal lymphatic function in chronic cases [6–8]. By contrast, surgical lymphatic reconstruction has become a therapeutic mainstay for advanced lymphedema. In particular, due to the advancements of microsurgical techniques, vascularized lymph node transfer (VLNT) has emerged as a promising physiological treatment aimed at restoring lymphatic continuity [9–12]. In VLNT, functional lymph nodes are implanted into the affected limb to re-establish lymphatic outflow pathways and immune function [13, 14]. Clinical and preclinical studies have proved that VLNT significantly reduces limb volume, alleviates fibrosis, decreases cellulitis prevalence, and improves patients’ quality of life [1, 15–20]. In animal models, VLNT has been shown to reconstitute lymphatic channels, enhance lymphangiogenesis, and even restore systemic immune responses by enabling dendritic cell trafficking and high endothelial venule (HEV) maintenance [21]. These findings have made VLNT the preferred surgical option for refractory secondary lymphedema.

Despite these advances, the therapeutic outcomes of VLNT vary. Factors such as the degree of preexisting lymphatic damage, donor site morbidity, and age-related lymph node degeneration may limit its efficacy [21, 22]. Several theories have been proposed to explain the mechanisms of VLNT: the “*lymph node pump theory*” suggesting that transplanted lymph nodes actively suck in and pump out lymph fluid to restore local lymphatic flow; the “*growth factor theory*” proposing that transplanted lymph nodes secrete lymphangiogenic factors such as VEGF-C to promote lymphatic regeneration; and a combined mechanism involving both active lymphatic pumping and growth factor–mediated lymphangiogenesis [23, 24]. Previous studies indicate that VLNT’s therapeutic efficacy crucially depends on the successful reconnection of lymphatic vessels and the maintenance of transplanted lymph node’s intrinsic functions [25, 26]. For example, Ishikawa et al. [21] reported that functional preservation of transplanted lymph nodes occurs only when the afferent lymphatic reconnection is achieved, which allows the maintenance of high endothelial venules (HEVs), the key conduits for lymphocyte homing and immune surveillance. However, achieving sufficient lymphangiogenesis and preserving immune competence in transplanted nodes remain challenging. Meanwhile, advances in regenerative medicine have highlighted the therapeutic potential of adipose tissue and its derivatives in the management of lymphedema. Recent studies have explored the adjunctive use of adipose-derived stem cells (ADSCs) to improve VLNT outcomes, as these cells secrete lymphangiogenic factors such as VEGF-C and VEGFR-3 which promote lymphatic regeneration [27–29]. While ADSC-assisted VLNT has shown synergistic effects in animal models, the clinical translation of cell-based therapies is limited by concerns regarding tumorigenicity, immunogenicity, and regulatory hurdles [30]. Other cell-free strategies, such as direct delivery of recombinant growth factors (e.g., VEGF-C) or conditioned medium from cultured ADSCs, have shown pro-lymphangiogenic effects. However, they face limitations including short protein half-life, high production costs, and batch-to-batch variability. In contrast, adipose liquid extract (ALE) is a physically prepared, cell-free derivative that retains a native milieu of angiogenic and lymphangiogenic factors, including VEGF, HGF, bFGF, and TGF-β1 [31–33]. However, whether ALE can enhance the functional and regenerative efficacy of VLNT in lymphedema has hitherto remained unexplored.

In this study, we investigate for the first time whether ALE supplementation enhances VLNT’s efficacy. Using a rat hindlimb lymphedema model, we combine standard pedicled VLNT with local injection of ALE at the transplant site. We then evaluate the effects on lymphatic regeneration, lymph node viability, and overall lymphedema reduction. Our aim is to ascertain whether ALE does synergistically function with VLNT to accelerate lymphatic repair and improve limb recovery, thus providing a novel strategy bolstering lymphedema surgery.

## Methods

### Animals

All animal procedures were approved by the Institutional Animal Care and Use Committee (IACUC) of Zunyi Medical University for the project entitled “Mechanism and clinical study of metformin targeting the AMPK/mTOR pathway in the treatment of secondary lymphedema” (Approval No. [2022]1-169; approval date 8 March 2022). All experiments were conducted in strict accordance with the institutional guidelines for animal research and the Guide for the Care and Use of Laboratory Animals (8th edition, National Academies Press). Female Sprague–Dawley rats (n = 54), aged 6–8 weeks and weighing 280–320 g, were purchased from Hunan SJA Laboratory Animal Co., Ltd. (Changsha, China). Female animals were selected to minimize confounding factors related to hormonal cycles and to simulate the clinical predominance of lymphedema in female breast cancer survivors. All rats were housed under pathogen-free conditions in a temperature- and humidity-controlled environment with a 12-h light/dark cycle and had free access to standard chow and water. Animals were acclimatized for one week before the experiments. The design, conduct and reporting of all animal experiments followed the ARRIVE 2.0 guidelines.

### Establishment of the lymphedema model

**Fig. 1:**
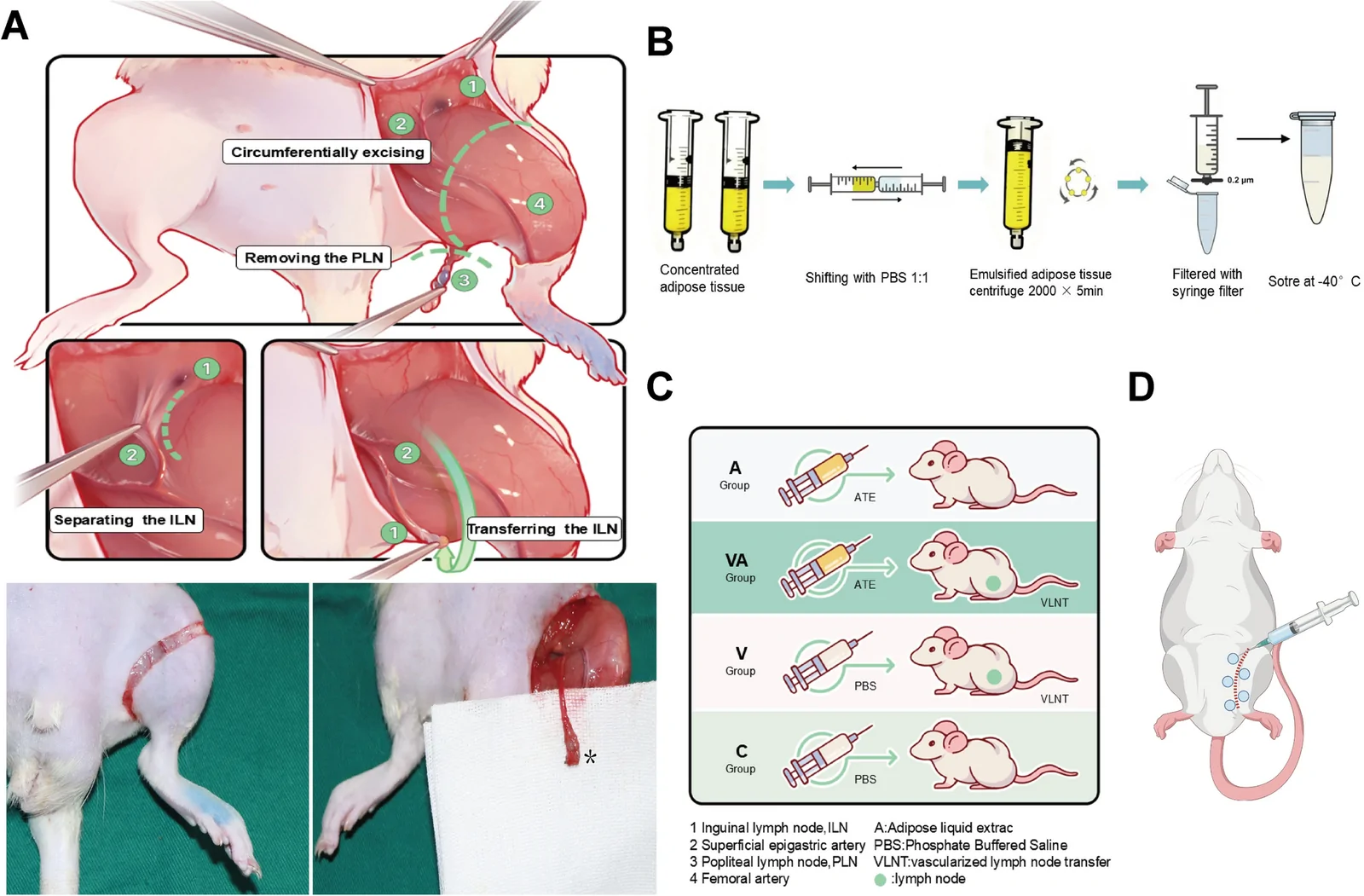
Rat hindlimb lymphedema model. VLNT schematic; ALE preparation; and grouping overview. **A** Schematic diagram of the hindlimb lymphedema model in SD rats. The illustration and intraoperative photographs show the excision of the popliteal lymph nodes and the transfer of the vascularized inguinal lymph node flap. The asterisk (*) indicates the vascularized lymph node transfer. **B** Flowchart illustrating the preparation process of adipose liquid extract (ALE) from adipose tissue. **C** The experimental design included four groups: the control group (C group), flap transfer without lymph nodes plus PBS injection; the ALE group (A group), flap transfer without lymph nodes plus ALE injection; the VLNT group (V group), vascularized lymph node transfer (VLNT) plus PBS injection; and the combined group (VA group), VLNT plus ALE injection. **D** Schematic illustration of the injection protocol. A total of 200 μL of ALE or PBS was divided equally into five subcutaneous injection sites (approx. 40 μL per site) around the transferred lymph node (marked by asterisk) and along the flap-recipient interface. Injections were performed daily on postoperative days 1, 2, and 3

Rats were anesthetized with 2.5% isoflurane in oxygen using a small animal anesthesia machine (induction in a chamber followed by maintenance via a nose cone). Next, the hair around the surgical site was removed, and the skin was disinfected. To establish the lymphedema model, 0.2 mL of 1% Evans Blue dye was injected subcutaneously into the dorsal paw to visualize the lymphatic vessels and popliteal lymph nodes. Fifteen minutes later, an incision was made at the ipsilateral popliteal fossa, and the blue-stained lymph nodes and lymphatic vessels were exposed by blunt dissection. The stained lymph nodes were completely excised, and the deep lymphatic vessels were ligated to block lymphatic drainage. After modeling, rats were randomly divided into two surgical groups to receive flap transfers either with or without lymph nodes. A skin flap based on the inferior epigastric artery was raised toward the inguinal region. For the VLNT flap, the inguinal lymph nodes were preserved, while for the control flap, the lymph nodes were removed (Fig. 1A). The vascularized or non-vascularized flaps were then transferred to the popliteal region and fixed to the surrounding muscles. The skin incision was closed with separate sutures. All rats were housed individually after surgery and monitored daily until recovery.

### Standardization and preparation of cell-free adipose liquid extract (ALE)

Human adipose tissue used in this study was obtained from discarded lipoaspirate donated by patients undergoing liposuction procedures. The study protocol was approved by the Biomedical Research Ethics Committee of the Affiliated Hospital of Zunyi Medical University (Approval No. KLLY-2022-044; approval date 31 December 2022). The collection and use of human tissue were conducted in strict accordance with the Declaration of Helsinki. Prior to tissue collection, all donors provided written informed consent. We employed a purely physical, mechanical emulsification method to generate a cell-free extract, ensuring clinical safety and ease of translation as described in our earlier study [31]. Briefly, fresh lipoaspirates were washed extensively with phosphate-buffered saline (PBS) to remove blood and debris. The clean adipose tissue was mixed 1:1 with PBS and mechanically emulsified by shifting between two 10 mL syringes connected via a Luer-lock connector (30 passes at a flow rate of 10 mL/s). The emulsion was then centrifuged at 2000×*g* for 5 min to separate the oil, aqueous, and pellet phases. The middle aqueous layer, rich in extracellular fluid and cytosolic proteins, was collected and passed through a 0.22-μm syringe filter to remove any residual cellular components and debris, yielding the final cell-free ALE (Fig. 1B). Total protein concentration was quantified using a BCA protein assay to ensure batch consistency. ALE aliquots were stored at – 40 °C until intraoperative use. As previously characterized by label-free LC-MS/MS and ELISA [31], ALE contains 1,742 quantified human proteins, among which 67 are angiogenesis-related and 25 are adipogenesis-related. Key growth factors detected include VEGF, bFGF, HGF, TGF-β1, EGF, and PDGF.

### Surgical treatment and administration protocol

Forty-eight rats with established lymphedema were randomized into four therapeutic groups (Fig. 1C): (1) Control (C group, n = 12): non-vascularized flap transfer + PBS vehicle; (2) ALE group (A group, n = 12): non-vascularized flap + ALE injection; (3) VLNT group (V group, n = 12): vascularized lymph node transfer + PBS vehicle; and (4) Combined group (VA group, n = 12): VLNT + ALE injection. To mimic a clinical regenerative therapy regimen, 200 μL of ALE (or PBS vehicle) was administered locally into the perinodal and flap implantation site on postoperative days 1, 2, and 3. Specifically, using a 30-gauge needle, the 200 μL volume was divided equally into five subcutaneous injection sites around the transferred lymph node and along the flap-recipient bed interface (approximately 40 μL per site, injected 1–2 mm deep). The injection sites were arranged as illustrated in Fig. 1D. This timing was chosen to support the critical early phase of graft integration and angiogenesis. The volume of 200 μL was chosen based on our previous dose-finding study in a murine wound model, in which 200 μL of ALE showed optimal efficacy without local toxicity [31]. According to ELISA quantification in that study, each 200 μL of ALE contains approximately 138 pg of bFGF, 13 pg of EGF, 286 pg of TGF-β1, 87 pg of VEGF, 94 pg of HGF, and 34 pg of PDGF. For the VLNT groups (V and VA), the inguinal lymph node flap was harvested from the same rat during surgery (autologous transfer); thus, donor nodes were obtained from 6- to 8-week-old rats, i.e., young healthy nodes. For the evaluation of age-related lymph node phenotypes, inguinal lymph nodes were also harvested from separate cohorts of young (6 months) and aged (20 months) Sprague–Dawley rats.

### Edema evaluation by limb circumference measuring

The extent of hindlimb swelling was evaluated by photographic documentation and circumference measurements. Representative pictures of the operated hindlimbs were taken immediately after surgery (day 0), and on postoperative days 3 and 14 using a digital camera to record morphological changes and edema resolution. The limb circumference was measured every 3 days after surgery until day 14 to assess the degree of swelling. All measurements were conducted under light isoflurane anesthesia, with the rats in a fully relaxed position. The circumference was measured at a consistent, pre-marked site on each hindlimb to ensure accuracy and reproducibility. The investigator performing the measurements was blinded to the group allocation.

### Indocyanine green (ICG) lymphography

Lymphatic drainage was assessed by ICG lymphography under isoflurane anesthesia. After depilation of the affected hindlimb, a total of 0.1 mL of 1.25 mg/mL ICG solution (Dandong Yichuang Pharmaceutical Co., Ltd., Dandong, China) was injected subcutaneously into the distal region of the affected limb on postoperative days 3 and 14 (n = 3/group). The migration of ICG from the injection site was visualized using a hand-held near-infrared fluorescence imaging device (Shinevia, Changsha, China) to trace lymphatic flow and assess functional recovery of the lymphatic system.

### Histological and immunofluorescence analysis

Rats were deeply anesthetized with 3–4% isoflurane in oxygen and euthanized by cervical dislocation. Death was confirmed by the absence of heartbeat and corneal reflex before tissue harvest. Skin tissues (1 cm × 1 cm) from the lymphedematous hindlimbs were collected on postoperative days 3 and 14. Lymph node grafts from the V and VA groups were also harvested on day 14 (n = 3/group). Inguinal nodes from 6 and 20 Mo rats were harvested at the designated ages. All samples were fixed in 4% paraformaldehyde at room temperature for 36 h, embedded in paraffin, and sectioned at 5 μm thickness for hematoxylin–eosin (H&E), Masson’s trichrome, and immunofluorescence staining. For histological analysis, sections were deparaffinized, rehydrated, and stained with H&E or Masson’s Trichrome Stain Kit (G1340, Solarbio, Beijing, China) according to the manufacturer’s protocol. The stained slides were examined under a light microscope to evaluate tissue morphology and fibrosis. For immunofluorescence analyses, antigen retrieval was performed using citrate buffer (C1010, Solarbio), and non-specific binding was blocked with antibody diluent (A1800, Solarbio) for 30 min. Sections were incubated overnight at 4 °C with primary antibodies against LYVE-1 (1:200, ab14917, Abcam); CD31 (1:200, ab182981, Abcam); and MECA-79 (1:200, sc-19602, Santa Cruz). After washing, Alexa Fluor® 488–conjugated donkey anti-rabbit IgG (1:500, ab150061, Abcam) and Alexa Fluor® 594–conjugated goat anti-rabbit IgG (1:500, ab150080, Abcam) were used as secondary antibodies. Nuclei were counterstained with DAPI (Sigma, USA). Images were acquired on a fluorescence microscope and analyzed with FIJI ImageJ (NIH, USA).

### Angiogenic and lymphangiogenic induction ability of ALE in Vitro

Human umbilical vein endothelial cells (HUVECs; ATCC, USA) and human lymphatic endothelial cells (HLECs; Xiamen Immocell Biotechnology, China, Cat. No. IM-H480) were used to assess the pro-angiogenic and pro-lymphangiogenic effects of ALE, respectively. Both cell types were cultured in their respective recommended media (endothelial cell medium for HUVECs, EGM-2MV for HLECs). Tube formation assays were performed as follows: Matrigel (356,230; Corning, USA) was thawed at 4 °C overnight. Pre-cooled 24-well plates were coated with 100 μL/well of Matrigel diluted 1:1 in PBS and solidified at 37 °C for 30 min. Cells (1.5 × 10^5^ per well for HUVECs or HLECs) were seeded onto the Matrigel and treated with 1 mL of medium containing ALE and basal medium at a 1:1 ratio (ALE group) or with PBS and basal medium (Control). Tube formation was observed and photographed at 0, 3, 6, and 10 h using a Nikon E200 microscope. Tubular structures were quantified from six random fields per well using ImageJ. Each assay was performed in triplicate.

### Statistical analysis

Data are presented as mean ± standard deviation (SD). Statistical analyses were performed using SPSS (IBM Corp., Armonk, NY, USA, Version 29.0). One-way analysis of variance (ANOVA) was used to compare differences among groups at each time point. A value of* p* < 0.05 was considered as statistically significant.

## Results

### ALE combined with VLNT synergistically alleviates hindlimb lymphedema in rats

**Fig. 2:**
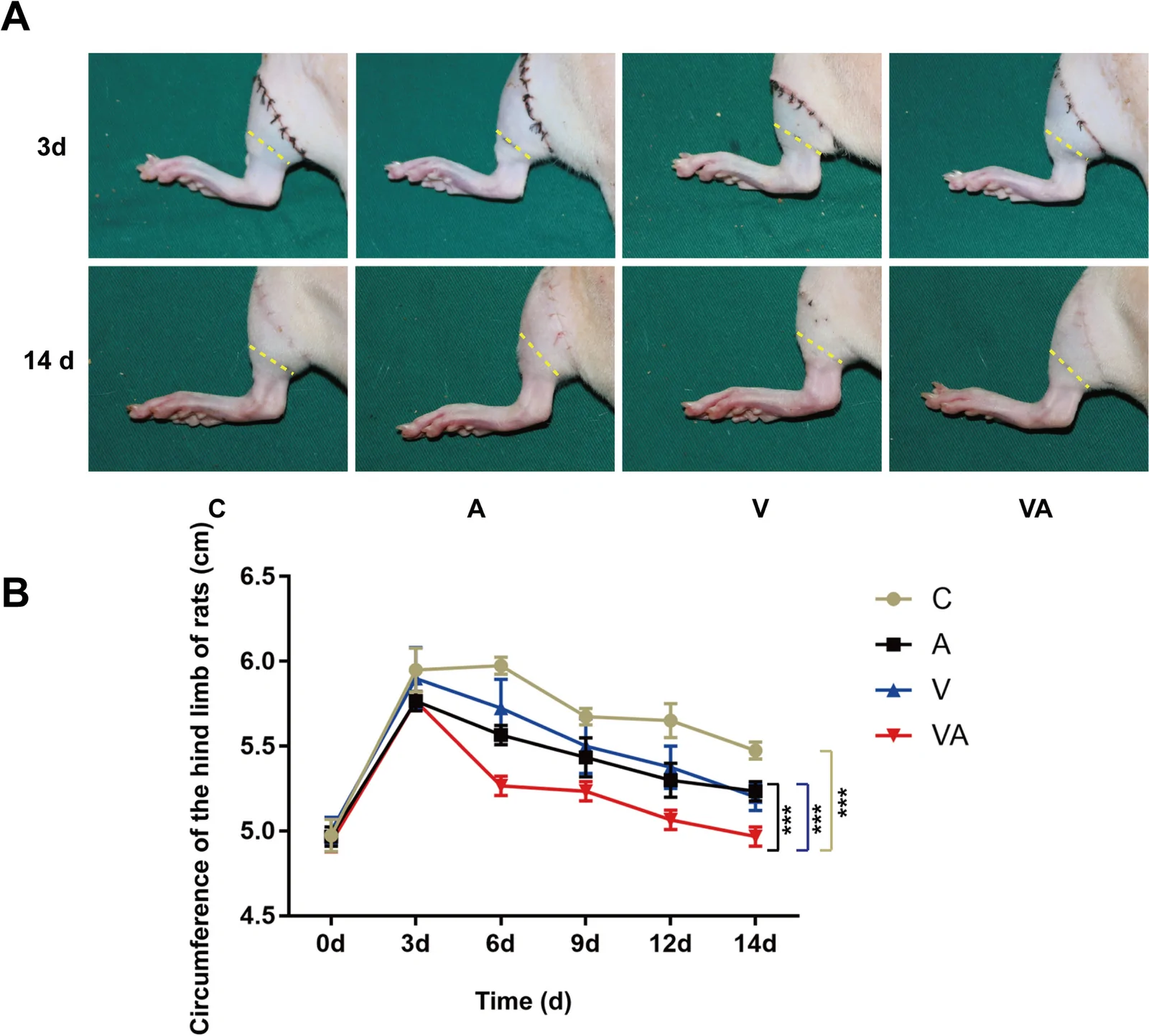
ALE combined with VLNT synergistically reduces hindlimb circumference and hence lymphedema in rats. **A** Representative hindlimb images on postoperative days 3 and 14. All groups showed decreased edema on day 14 relative to day 3, with VA displaying the most pronounced reduction, a visible Achilles tendon, and a smoother skin. The circumference measurement site is indicated by a yellow dashed line. **B** Quantitative analysis of hindlimb circumference. Circumference values were comparable among groups immediately after surgery (day 0), peaked on day 3, and progressively decreased thereafter. The VA group showed the fastest reduction (from 5.77 ± 0.03 cm on day 3 to 4.97 ± 0.03 cm on day 14), while C showed the minimum improvement; V and A showed moderate reductions. On day 14, VA differed significantly from C, V, and A (*p* < 0.05, ***p ≤ 0.001, n = 3)

We first evaluated the gross therapeutic efficacy of the combined strategy on limb swelling. While edema peaked on day 3 across all groups due to surgical trauma, the trajectory of resolution differed significantly. The VA group (VLNT + ALE) exhibited the most rapid and pronounced regression of swelling. By day 14, hindlimbs in the VA group displayed near-normal morphology with clearly visible Achilles tendons and smooth skin contours (Fig. 2A). Quantitative circumference analysis confirmed a synergistic effect: the VA group showed a significantly greater reduction (final circumference 4.97 ± 0.03 cm) compared to VLNT alone (5.20 ± 0.04 cm), ALE alone (5.23 ± 0.03 cm), or Control (5.47 ± 0.03 cm) (*p* < 0.001; Fig. 2B). These data suggest that ALE acts as a potent accelerator for volume reduction when combined with physiological reconstruction.

### ALE combined with VLNT reduces skin thickness in lymphedematous hindlimbs

**Fig. 3:**
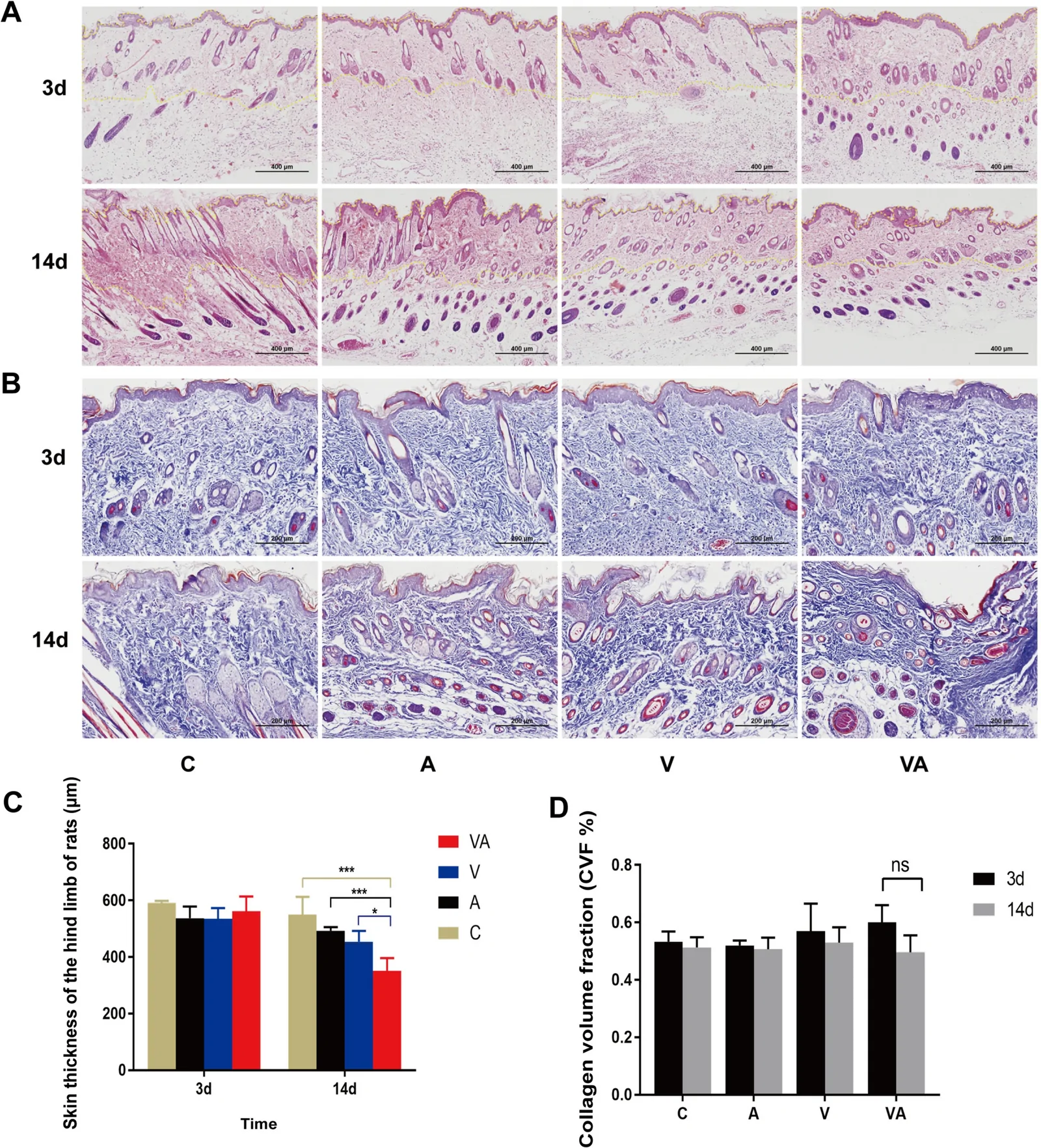
ALE combined with VLNT reduces skin thickness but does not change CVF% in the lymphedematous hindlimb of rats. **A**, **B** Representative H&E images on days 3 and 14. Skin thickness was measured as the distance from the epidermis to the upper margin of the fat layer (yellow dashed lines). On day 3, thickness was comparable among groups. On day 14, values were 549.74 ± 36 μm (**C**), 493.23 ± 7.3 μm (**A**), 454.10 ± 22.05 μm (V), and 350.77 ± 26.39 μm (VA). VA was significantly thinner than all other groups (*p < 0.05, ***p ≤ 0.001; n = 3). Scale bar = 400 μm. Images were processed in FIJI ImageJ. **C**, **D** Representative Masson’s trichrome images (blue collagen). CVF% values were comparable among groups at both time points (*p* > 0.05, n = 3), although VA showed a more organized collagen arrangement on day 14. Scale bar = 200 μm. “ns” indicates no statistical difference. Images were processed in FIJI ImageJ

Increased skin thickness and collagen deposition are hallmarks of secondary lymphedema, whereas reductions in these parameters indicate therapeutic benefit. To evaluate tissue remodeling, skin samples were examined on postoperative days 3 and 14. Skin thickness was defined as the average distance from the epidermis to the upper margin of the subcutaneous fat layer (yellow dashed lines; Fig. 3A). On day 3, no significant differences were observed among groups. By day 14, however, skin in VA appeared thinner (350.77 ± 26.39 μm) than in C (549.74 ± 36 μm), A (493.23 ± 7.3 μm), and V (454.10 ± 22.05 μm). There was no significant difference between A and C (*p* = 0.15), indicating that ALE alone did not markedly affect skin thickness. In contrast, V differed from C (*p* = 0.028), demonstrating that VLNT alleviated dermal thickening. Furthermore, VA was significantly thinner than both V (*p* = 0.02) and C (*p* = 0.001), suggesting that the combined treatment of ALE and VLNT more effectively reduced lymphedema-associated skin thickening (Fig. 3B).

Masson’s trichrome staining was used to evaluate collagen deposition. Collagen fibers were widely distributed in the dermis and subcutaneous tissue of all groups at both time points, with no significant differences in per cent collagen volume fraction (CVF%). Nevertheless, on day 14, collagen in the VA group exhibited no change in CVF% but a more organized and regular alignment than the other groups did, which may be associated with the synergistic effects of ALE and VLNT (Fig. 3C and D).

### ALE orchestrates functional lymphatic network reconstruction and eliminates dermal backflow

**Fig. 4:**
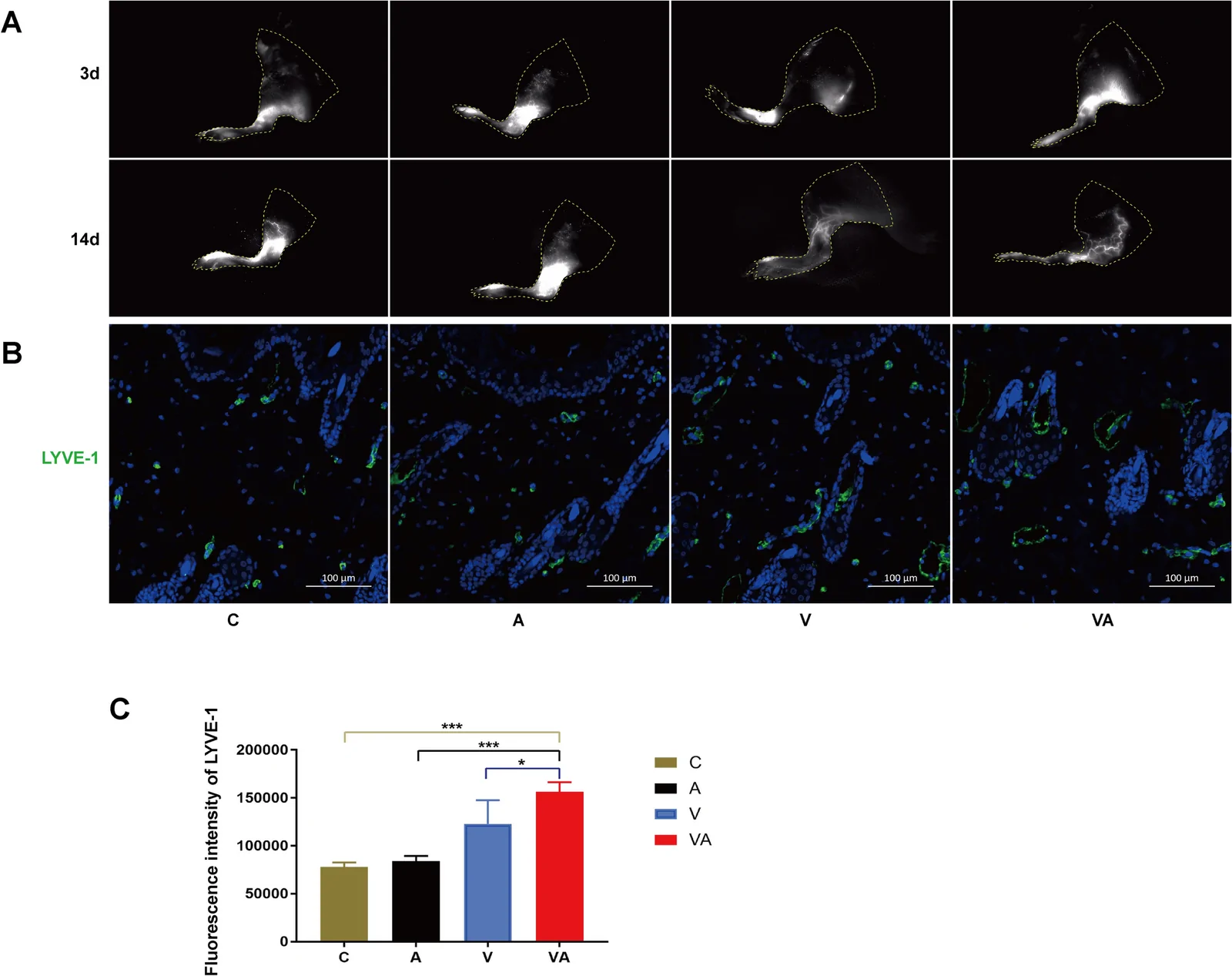
ALE synergistically enhances the lymphangiogenic effect of VLNT in the lymphedematous hindlimbs of rats. **A** ICG lymphography was performed to evaluate lymphatic drainage in the lymphedema rat model. On day 3, all groups exhibited dermal backflow of lymphatic fluid that accumulated in the distal hindlimb, with no visible linear lymphatic vessels connecting the distal and proximal regions. By day 14, extensive dermal backflow persisted in the distal limbs of the C and A, and no continuous lymphatic vessels were detected connecting the distal to the proximal limb. In contrast, V and VA developed newly formed linear lymphatic vessels bridging the distal and proximal regions. Mild dermal reflux was still present in the V group, whereas the VA group exhibited continuous and patent lymphatic channels without any apparent dermal backflow. **B**, **C** LYVE-1 immunofluorescence was performed on day 14 to assess lymphangiogenesis in the skin of the operated hindlimb. Consistent with the ICG lymphography findings, C and A exhibited a low LYVE-1^+^ expression without circular organization, whereas V and VA showed higher LYVE-1^+^ signals, with a small portion of LYVE-1^+^ lymphatic endothelial cells forming circular structures in the V group and the majority forming circular aggregates in the VA group. Quantitative fluorescence analysis (FIJI ImageJ) showed the VA exhibited significantly higher LYVE-1^+^ fluorescence intensity as compared to the other groups (*p* < 0.05, ***p ≤ 0.001; n = 3). Scale bar = 100 μm

Beyond simple volume reduction, the restoration of functional lymphatic drainage is paramount. ICG lymphography on day 14 revealed that while Control and ALE-only groups suffered from persistent fluid stagnation and extensive dermal backflow, the VLNT group showed partial recovery with some linear flow but residual reflux. Strikingly, the VA group showed the appearance of continuous, well-defined linear lymphatic drainage pathways bridging the distal and proximal limb, with no observable dermal backflow (Fig. 4A).

This functional recovery was underpinned by robust structural regeneration. Immunofluorescence analysis of LYVE-1 demonstrated that the VA group not only possessed a higher density of lymphatic vessels but, more importantly, formed organized, circular lymphatic capillary networks rather than the scattered, disorganized endothelial cells seen in controls (Fig. 4B). Quantitative analysis confirmed that the combination of ALE and VLNT significantly upregulated LYVE-1 expression compared to VLNT alone (*p* = 0.018) (Fig. 4C), indicating that ALE potentiates the lymphangiogenic integration of the lymph node graft.

### By promoting angiogenesis ALE enhances the therapeutic efficacy of VLNT in lymphedema

**Fig. 5:**
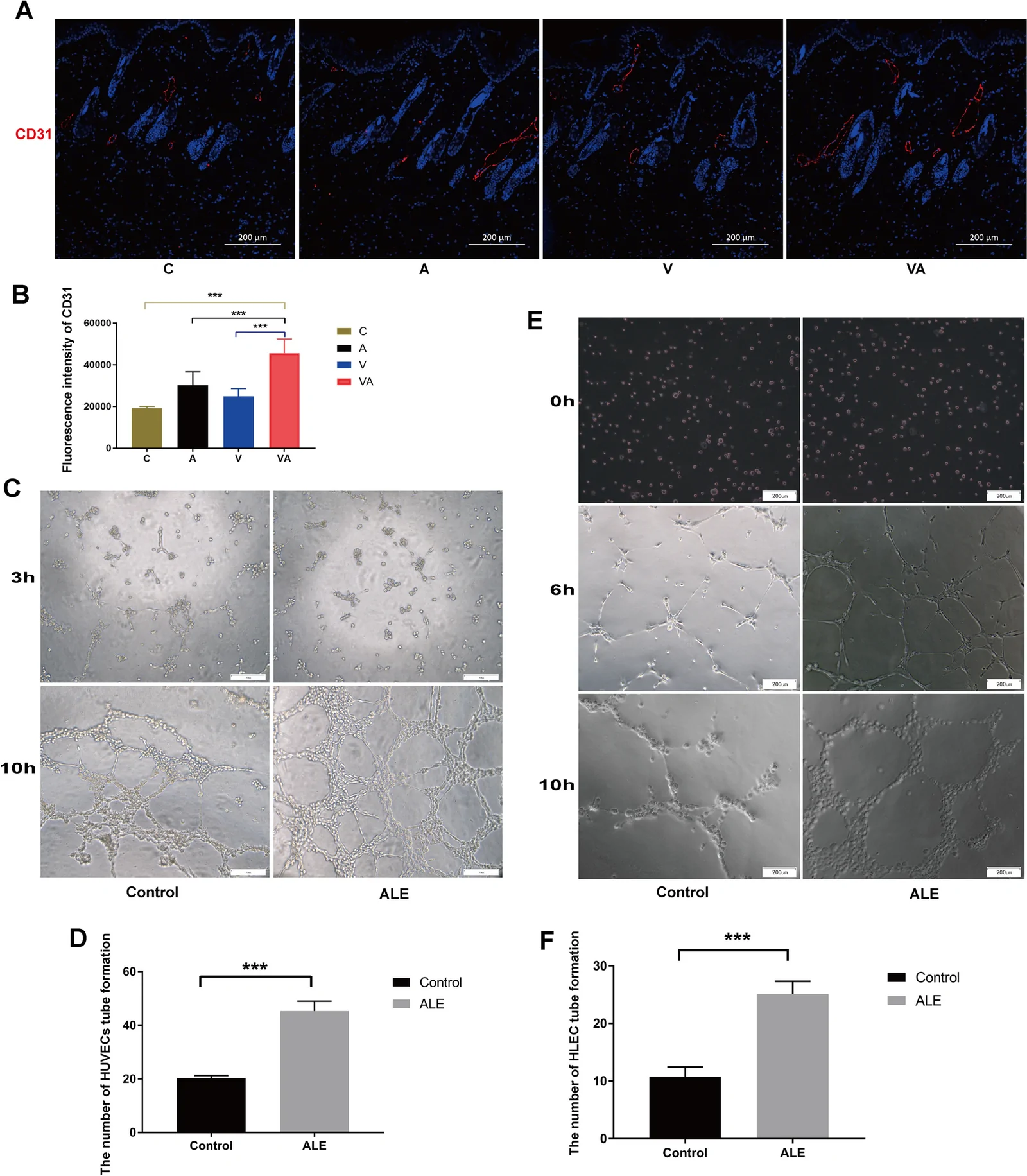
ALE treatment enhances VLNT efficacy by promoting angiogenesis and lymphangiogenesis. **A**, **B** CD31 (endothelial cell marker) immunofluorescence was performed in skin on day 14 to evaluate vascular regeneration. Fewer CD31^+^ endothelial cells were detected in the C and V groups, whereas higher numbers of CD31^+^ endothelial cells with evident tubular structures were observed in the A and VA groups. Quantitative analysis (FIJI ImageJ) revealed that the VA group exhibited the highest CD31 fluorescence intensity, followed by the A group. The fluorescence intensity in the VA group was significantly higher than that in the other groups (*p* < 0.05, ***p ≤ 0.001; *n* = 3). Scale bar = 200 μm. **C**, **D** Human umbilical vein endothelial cells (HUVECs) tube formation with PBS (control) or ALE. Images were captured 3 h and at 10 h after treatment. At 10 h, the control group showed partial formation of circular tubular structures, whereas the ALE-treated cells displayed more circular and highly branched tubular networks. Quantitative analysis (FIJI ImageJ) showed that the number of tubular structures was 20.33 ± 0.38 in the C group and 45.33 ± 1.5 in the ALE group, indicating a significant difference (*p* < 0.001, ***p ≤ 0.001; n = 6). Scale bar = 200 μm. **E**, **F** Human lymphatic endothelial cells (HLECs) tube formation with PBS (Control) or ALE. Images were captured at 0, 6, and 10 h after treatment. At 10 h, the control group showed scattered and poorly connected tube-like structures, whereas the ALE-treated cells formed extensive, highly branched tubular networks. Quantitative analysis (FIJI ImageJ) showed that the number of tubular structures was 10.77 ± 1.67 in the control group and 25.15 ± 2.16 in the ALE group, indicating a significant difference (*p* < 0.001, ***p ≤ 0.001; n = 6). Scale bar = 200 μm

Graft survival and function rely on the rapid re-establishment of blood supply [21, 34, 35]. Given the known angiogenic content of adipose extracts, we investigated whether ALE enhances vascularization in the VLNT setting. CD31 staining on day 14 revealed a sparse vascular network in the Control and VLNT-only groups. In contrast, the ALE-treated groups (A and VA) exhibited dense, mature vascular networks with abundant circular microvessels (Fig. 5A). The VA group showed the highest angiogenic response, significantly superior to VLNT alone (*p* = 0.001) (Fig. 5B). In vitro HUVEC tube formation assays corroborated these findings, where ALE treatment doubled the number of tubular structures compared to controls (*p* < 0.0001) (Fig. 5C and D). To assess the pro-lymphangiogenic effect of ALE, we performed tube formation assays using human lymphatic endothelial cells (HLECs). ALE treatment markedly promoted HLECs tube formation compared to the control group (Fig. 5E). Quantitative analysis showed that the number of tubular structures in the ALE-treated group was 25.15 ± 2.16, significantly higher than that in the control group (10.77 ± 1.67, *p* < 0.001) (Fig. 5F). These results provide direct evidence that ALE possesses pro-lymphangiogenic activity in vitro, complementing the in vivo findings of increased LYVE-1^+^ lymphatic vessel density and improved lymphatic drainage.

### ALE restores the immune niche by regenerating high endothelial venules (HEVs) in transferred nodes

**Fig. 6:**
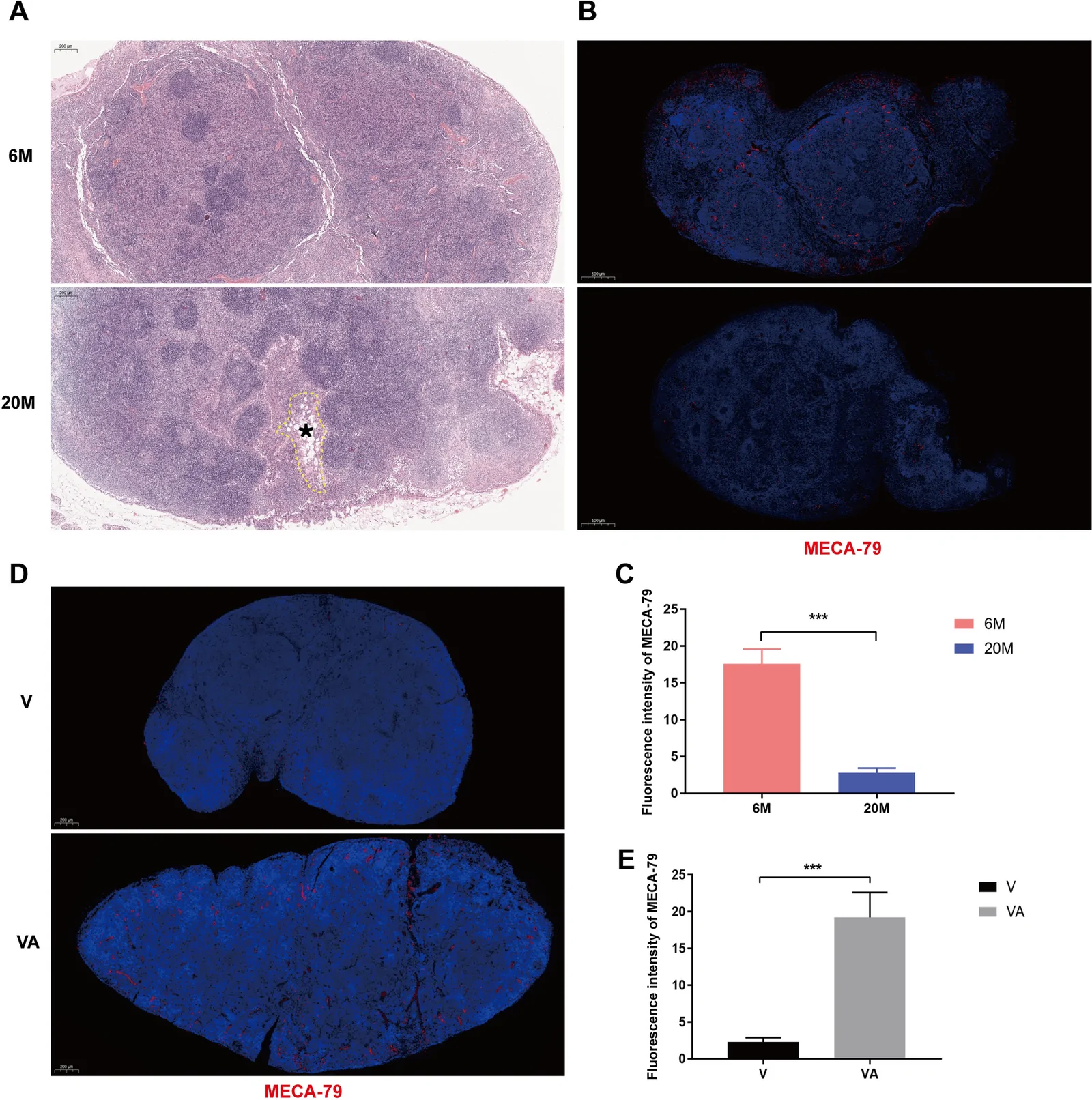
Transferred lymph nodes with ALE treatment and young lymph nodes show a higher MECA-79 expression. **A** H&E staining of inguinal lymph nodes from young (6 Mo) and aged (20 Mo) rats. The 6 Mo group shows a dense tissue with clear architecture, whereas the 20 Mo group exhibits a disorganized architecture with fatty infiltration inside the node. “*” denotes areas of fatty infiltration. Scale bar = 200 μm. **B** MECA-79 immunofluorescence in inguinal lymph nodes from 6 and 20 Mo rats. MECA-79 (red) is more abundant in the 6 Mo than in the 20 Mo nodes. n = 3. Scale bar = 500 μm. **C** MECA-79 immunofluorescence in transferred lymph nodes on day 14. The VA group shows markedly higher MECA-79 expression than the V group, which displays only scant red fluorescence. n = 3. Scale bar = 200 μm. Groups C and A are not shown in (**C**) because they did not receive lymph node transfer; their normal inguinal nodes are presented as young controls in (**A**, **B**). **D** Quantification of MECA-79 fluorescence intensity analyzed in FIJI ImageJ. 6 Mo vs. 20 Mo: *p* = 0.001; V vs. VA: *p* = 0.001; 6 Mo vs. VA: *p* = 0.514; 20 Mo vs. V: *p* = 0.395. *** indicates *p* ≤ 0.001; ns indicates *p* > 0.05

High endothelial venules (HEVs) in lymph nodes are the primary portals for lymphocyte entry from the bloodstream and are essential for maintaining lymph node microarchitecture and immune activity. Accordingly, the presence of HEVs is a hallmark of a functional, immunologically competent lymph node [36, 37]. We hypothesized that VLNT might suffer from HEV regression due to ischemic injury and that ALE could rescue this phenotype. We first established an age-related baseline, finding that “Young” (6 Mo) healthy nodes possess abundant MECA-79^+^ HEVs, whereas “Aged” (20 Mo) nodes show significant HEV loss and fatty degeneration (*p* = 0.001) (Fig. 6A and B).

Remarkably, in our transplant model, lymph nodes in the VLNT-alone group (V group) exhibited a MECA-79 expression pattern resembling the “Aged” phenotype, indicating functional regression. However, the addition of ALE (VA group) dramatically rescued HEV density (Fig. 6C). Quantitative analysis showed that MECA-79 expression in the VA group was significantly higher than in the V group (*p* = 0.001) and statistically indistinguishable from healthy “Young” nodes (*p* = 0.514) (Fig. 6D). This indicates that ALE treatment preserves the structural integrity of HEVs, a key component of the lymph node’s immune microarchitecture, to a level similar to that observed in young nodes.

## Discussion

In this study, we demonstrate that the local application of Adipose Liquid Extract (ALE) acts as a potent biological adjuvant that significantly potentiates the therapeutic efficacy of Vascularized Lymph Node Transfer (VLNT). While VLNT alone provides a physiological bridge for lymphatic drainage [9, 24], our data show that its combination with ALE results in a synergistic acceleration of edema resolution (Fig. 2) and tissue remodeling (Fig. 3). Distinct from previous approaches using expanded stem cells [30, 38], we utilized a cell-free strategy that can be prepared in real-time in the operating room. Our findings reveal that this “combined technology” works not merely by adding two therapies together, but by fundamentally improving the recipient bed’s microenvironment. Specifically, it orchestrates robust angiogenesis, lymphangiogenesis (Figs. 4 and 5), and the critical restoration of High Endothelial Venules (HEVs) within the transferred nodes (Fig. 6).

The establishment of a functional vascular network is the prerequisite for lymph node graft survival and subsequent lymphangiogenesis. This “vascular-lymphatic coupling” is central to our observed results. The transplanted lymph node can be viewed as the “seed,” while the recipient bed acts as the “soil.” In chronic lymphedema, the recipient site is often fibrotic and ischemic, creating a hostile environment for the graft [3, 22]. ALE, rich in bioactive factors such as VEGF, bFGF, and HGF [31, 33], effectively “fertilizes” this soil. Our CD31 (Fig. 5A and B) and LYVE-1 (Fig. 4B and C) data confirm that ALE promotes a dual-regenerative process. Angiogenesis provides the necessary perfusion to metabolically support the graft, while lymphangiogenesis physically reconnects the graft to the host lymphatic system. This functional reconnection is supported by the appearance of continuous linear drainage channels without dermal backflow on ICG lymphography (Fig. 4A). The direct pro-lymphangiogenic activity of ALE was further confirmed by HLECs tube formation assays (Fig. 5E, F). This creates a favorable niche that prevents the atrophy of the transferred node, a common cause of VLNT failure in clinical settings [21, 25]. We acknowledge that ICG lymphography provides functional evidence of improved lymphatic flow rather than direct anatomical proof of vessel anastomosis; direct visualization would require microlymphangiography or histological tracing, which were not performed in this study.

A pivotal finding of our study is the ALE-mediated restoration of MECA-79^+^ HEVs in transplanted lymph nodes. HEVs are specialized post-capillary venules that serve as the primary gateways for lymphocyte entry into the lymph node parenchyma. They are essential for maintaining immune surveillance [36, 37]. Surgical transfer and ischemia often lead to HEV regression, transforming the lymph node into a non-functional fibrotic scar. We observed that VLNT alone—resembling the “aged” phenotype observed in 20-month-old rats (Fig. 6A and B)—failed to maintain high HEV density. In contrast, ALE treatment preserved HEV structural integrity at levels comparable to young, healthy nodes (Fig. 6C and D). This suggests that ALE supports the structural basis of the immune niche in transplanted nodes. We acknowledge that the conclusion of HEV restoration is based solely on MECA-79^+^ density, and additional markers of lymph node aging or immune function (e.g., fibrotic deposition, T-cell zone integrity, lymphocyte trafficking assays) were not examined. Preserving the structural integrity of HEVs is clinically relevant, as it may help reduce the frequency of recurrent cellulitis, a major complication in lymphedema patients [20, 39].

From a translational perspective, the cell-free nature of ALE offers distinct advantages over adipose-derived stem cell (ADSC) therapies. Although ADSCs are effective [28–30], their clinical application is restricted by several hurdles. These include the need for Good Manufacturing Practice (GMP) facilities, prolonged culture periods, potential tumorigenicity risks, and complex regulatory approval processes [40]. ALE, as a cell-free extract, circumvents these hurdles. It retains the potent secretome of adipose tissue—including extracellular vesicles and soluble cytokines [31, 32]—while eliminating cellular components. The preparation of ALE is purely physical (emulsification and centrifugation), requiring no enzymatic digestion or xenogeneic additives, and takes less than 30 min (Fig. 1B). This “point-of-care” capability allows surgeons to harvest fat, prepare ALE, and administer it within a single surgical procedure, making it a highly translatable strategy for immediate clinical adoption.

We acknowledge that the use of human-derived ALE in a rat model may raise concerns about potential xenogeneic immune reactions. However, ALE is a cell-free, physically processed extract that contains no cellular antigens; its major components (e.g., growth factors, extracellular matrix fragments) are highly conserved across mammalian species [31]. Importantly, our previous study using human ALE in a murine wound healing model observed no overt local inflammation in ALE-treated animals (e.g., erythema, purulent discharge, or delayed healing) [31]. Similarly, other studies using human acellular dermal matrix or platelet lysates in rodent models have reported minimal immunogenicity, attributed to the absence of intact cells and the evolutionary conservation of key bioactive proteins [41]. Nevertheless, we cannot completely rule out mild, subclinical immune reactions that might theoretically influence the results. For clinical translation, autologous ALE (prepared from the patient’s own adipose tissue) would completely avoid this issue, as discussed in our prior work [31].

It is important to recognize the limitations of this study. The follow-up period was 14 days, which captured only the early phase of graft integration and lymphedema regression. While ALE remarkably enhanced edema reduction (Fig. 2) and prevented skin thickening (Fig. 3), long-term studies are needed to assess the durability of HEV preservation and fibrosis prevention. Future investigations will employ spatial transcriptomics to further elucidate the molecular mechanisms by which ALE regulates the specialized differentiation of HEV endothelial cells.

In conclusion, cell-free ALE synergistically enhances the therapeutic efficacy of VLNT. It promotes vascular-lymphatic coupling and restores the structural integrity of the immune niche in transplanted nodes. As a readily available, safe, and cell-free biologic adjuvant, ALE represents a promising translational strategy to optimize surgical outcomes in secondary lymphedema.

## Data Availability

All data generated or analyzed during this study are included in this published article and its supplementary information files. Additional raw datasets are available from the corresponding author on reasonable request.
